# Expression of Androgen Receptor Splice Variants in Prostate Cancer Bone Metastases is Associated with Castration-Resistance and Short Survival

**DOI:** 10.1371/journal.pone.0019059

**Published:** 2011-04-28

**Authors:** Emma Hörnberg, Erik Bovinder Ylitalo, Sead Crnalic, Henrik Antti, Pär Stattin, Anders Widmark, Anders Bergh, Pernilla Wikström

**Affiliations:** 1 Department of Medical Biosciences, Pathology, Umeå University, Umeå, Sweden; 2 Department of Surgical and Perioperative Sciences, Orthopedics and Urology & Andrology, Umeå University, Umeå, Sweden; 3 Department of Chemistry, Umeå University, Umeå, Sweden; 4 Department of Radiation Sciences, Oncology, Umeå University, Umeå, Sweden; Virginia Commonwealth University, United States of America

## Abstract

**Background:**

Constitutively active androgen receptor variants (AR-V) lacking the ligand binding domain (LBD) may promote the development of castration-resistant prostate cancer (CRPC). The expression of AR-Vs in the clinically most important metastatic site, the bone, has, however, not been well documented. Our aim was therefore to compare levels of AR-Vs in hormone-naive (HN) and CRPC bone metastases in comparison to primary PC and non-malignant prostate tissue, as well as in relation to AR protein expression, whole-genome transcription profiles and patient survival.

**Methodology/Principal Findings:**

Hormone-naïve (n = 10) and CRPC bone metastases samples (n = 30) were obtained from 40 patients at metastasis surgery. Non-malignant and malignant prostate samples were acquired from 13 prostatectomized men. Levels of full length AR (ARfl) and AR-Vs termed AR-V1, AR-V7, and AR-V567es mRNA were measured with RT-PCR and whole-genome transcription profiles with an Illumina Beadchip array. Protein levels were examined by Western blotting and immunohistochemistry. Transcripts for ARfl, AR-V1, and AR-V7 were detected in most primary tumors and metastases, and levels were significantly increased in CRPC bone metastases. The AR-V567es transcript was detected in 23% of the CRPC bone metastases only. A sub-group of CRPC bone metastases expressed LBD-truncated AR proteins at levels comparable to the ARfl. Detectable AR-V567es and/or AR-V7 mRNA in the upper quartile, seen in 1/3 of all CRPC bone metastases, was associated with a high nuclear AR immunostaining score, disturbed cell cycle regulation and short survival.

**Conclusions/Significance:**

Expression of AR-Vs is increased in CRPC compared to HN bone metastases and associated with a particularly poor prognosis. Further studies are needed to test if patients expressing such AR-Vs in their bone metastases benefit more from drugs acting on or down-stream of these AR-Vs than from therapies inhibiting androgen synthesis.

## Introduction

Androgens regulate normal prostate tissue growth and differentiation, through activation of androgen receptors in epithelial and stroma cells, and also play an important role during all phases of prostate cancer (PC) growth. The standard therapy for patients with advanced PC is consequently to reduce androgens, either by surgical or medical castration. After a period of initial remission tumors eventually relapse, predominantly within the bone, and are then termed castration-resistant PC (CRPC). The mechanisms controlling CRPC growth in bone metastases are however largely unknown and seldom explored by actually examining such metastases in patients.

Interestingly, the androgen receptor (AR) is commonly active in CRPC despite castrate levels of circulating testosterone [1], [2] and intense nuclear AR immunostaining in CRPC bone metastases is related to a particularly short survival [3]. Different mechanisms have been suggested to contribute to AR activation in CRPC, including intracrine synthesis of androgens, genetic and epigenetic changes of the AR making it more sensitive to activation by androgens or other ligands [1], [4], [5] and the expression of constitutively active AR variants (AR-V, see below). These findings suggest that CRPC could be successfully treated with the novel drugs now in clinical trials that block androgen synthesis or AR in metastases (reviewed in [6]), but that constitutively active receptors could still be a problem. Studies are therefore needed to explore if these AR-Vs are commonly expressed in CRPC bone metastases or not.

Structurally the human *AR* gene is composed of eight exons encoding a 110 kDa protein consisting of a NH_2_ terminal trans-activation domain (NTD), a DNA binding domain (DBD), a hinge region, and the COOH terminal domain (CTD) which is also the ligand-binding domain (LBD) [7]. The LBD, encoded by exons 4–8, is not essential for transcriptional activity [8]. Truncated AR-Vs lacking the LBD and proposed to be constitutively active receptors have been detected in PC cell lines as well as in clinical samples, including benign, malignant and metastatic tissue, as results from alternative splicing or non-sense mutations of the human *AR* gene [9], [10], [11], [12], [13], [14], [15], [16], [17]. Increased levels of AR splice variants have been detected in CRPC when compared to hormone-naïve (HN) PC [13], [14], [16]. Those studies included CRPC samples obtained from primary tumors at surgery, or soft–tissue metastases and a few bone metastases collected at autopsy. The expression of AR-Vs in the clinically most relevant metastatic site, the bone, has not been well documented.

The aim of this study was therefore to analyze expression of the full length AR (ARfl) and its clinically most abundant splice variants here termed AR-V1, AR-V7 [14] (also referred to as AR3 [13], [17], and AR-V567es [16] in HN and CRPC bone metastases in comparison to expression in primary PC and in non-malignant prostate tissue. Moreover, AR transcript levels were related to AR protein expression, whole-genome transcription profiles and clinical outcome.

## Materials and Methods

### Ethics statement

The study was approved by the local ethic review board of Umeå University and participants gave written or verbal consent.

### Patients

Bone metastases were obtained from a series of fresh-frozen and formalin fixed paraffin embedded biopsies collected from patients with PC operated for metastatic spinal cord compression or pathologic fractures at Umeå University Hospital (2003–2009). These patients have been thoroughly described in [3] and clinical characteristics and therapies are summarized in Table 1. The study also includes 13 patients who were treated with radical prostatectomy at Umeå University Hospital, between Feb 2005 and Sep 2006. Median age for the patients was 60 years (range 48–68 years) and median PSA were 6.6 ng/ml (range 3.5–24 ng/ml). Eleven of the patients had tumors graded as Gleason score (GS) 7 and two as GS 8. Four of the tumors were in stage T2 and nine in stage T3. Immediately after surgical removal the prostates were brought to the Pathology department and cut in 0.5 cm thick slices. From these slices 20 samples were punched from each prostate using a 0.5 cm steel cylinder and frozen in −70°C within 30 minutes after surgery. The prostate slices were then fixed in 4% formaldehyde for 24 h, dehydrated, embedded in paraffin, cut in 5 micron thick sections and stained with hematoxylin-eosin. The nature of the frozen tissue samples (non-malignant or malignant) was determined by their location in the whole-mount sections, but also verified by light microscopy of hematoxylin/eosin-stained cryostat section from each of the tissue samples analyzed as described below. In these sections the percentage of tumor tissue was quantified and the tumor GS determined.

**Table 1:** Clinical characteristics of patients with prostate cancer bone metastases obtained at orthopedic surgery.

Clinical characteristics	Hormone-naïve n = 10	Castration-resistant^a^ n = 30
**Age at PCa diagnosis** (yrs)	79 (60–85)	70 (51–86)
**Age at surgery** (yrs)	¨	73 (54–88)
**Serum PSA at diagnosis** (ng/ml)	156 (21–10000)	71 (2–2558)
**Serum PSA at surgery** (ng/ml)	¨	335 (4–5139)
**Androgen deprivation therapy prior to surgery**		
Castration^b^	-	20
Castration plus anti-androgen^c^	-	10
**Radiation prior to surgery** d		
Yes	0	4
No	10	26
**Chemotherapy prior to surgery**		
Yes	0	2
No	10	28
**Follow-up after surgery** e (months)	26 (0–66)	4.5 (0.25–35.50)

Continuous values are given as median (min–max values).

a Castration-resistant patients had disease progression after long-term androgen deprivation therapy.

b Includes surgical ablation, LHRH/GNRH agonist therapy, and therapy with anti-androgens (casodex, flutamide), but specifically no anti-androgens at the time for orthopedic surgery.

c Castration therapy as stated above and anti-androgen therapy (casodex) at the time for orthopedic surgery.

d Radiation towards operation site.

e The time between date of operation and the latest follow-up examination or death.

### Tissue preparation and RNA extraction

Representative areas of bone metastases, primary PC and non-malignant prostate tissue were cryo-sectioned into extraction tubes and RNA was isolated using the Trizol protocol (Invitrogen, Stockholm, Sweden). The percentage of tumor cells in the samples varied between 25–95%. The RNA concentrations were quantified by absorbance measurements using a spectrophotometer (ND-1000 spectrophotometer; NanoDrop Technologies, Inc., Wilmington, DE). The RNA quality was analyzed with the 2100 Bioanalyzer (Agilent Technologies, Santa Clara, CA, USA) and verified to have a RNA integrity number ≥6.

### Real time RT-PCR

Two-hundred (200) ng of RNA were reversed transcribed with Superscript II reverse transcriptase (Invitrogen) in a total volume of 10 µl. The subsequent PCR amplification was performed using the Biorad iQ5 iCycler (Bio-Rad Laboratories, Hercules, CA). Reactions were done in a 20 µl volume using the IQ SYBR Green Supermix (Bio-Rad laboratories). The following primer sets were used; ARfl: 5′-CCATCTTGTCGTCTTCGGAAATGTTATGAAGC-3′ and 5′-AGCTTCTGGGTTGTCTCCTCAGTGG-3′, AR-V1: 5′-CCATCTTGTCGTCTTCGGAAATGTTATGAAGC-3′ and 5′-CTGTTGTGGATGAGCAGCTGAGAGTCT-3′, AR-V7: 5′-CCATCTTGTCGTCTTCGGAAATGTTATGAAGC-3′ and 5′-TTTGAATGAGGCAAGTCAGCCTTTCT-3′, and AR-V567es: 5′- CCAAGGCCTTGCCTGATTGC and 5′-TTGGGCACTTGCACAGAGAT-3′, resulting in amplicons of 143, 145, respectively 125 bp, as previously described [14], [16]. Primers for the housekeeping gene RPL13A (5′-GTACGCTGTGAAGGCATCAA-3′ and 5′- GTTGGTGTTCATCCGCTTG-3′) amplified a 125 bp fragment. Each sample was run in duplicates and adjusted for corresponding RPL13A levels. Melting curve analysis confirmed the amplified products, which were also analyzed by 1% agarose gel electrophoresis to confirm the expected size (data not shown).

### Western blot analysis

Fresh-frozen bone metastases and prostate samples were cryo-sectioned and proteins were extracted from 20 sections (10 µm each) using the AllPrep Mini kit, according to the manufacturer's instructions (Qiagen, Hilden, Germany). Protein concentration was determined by the BCA Protein assay (Pierce Chemical Co., IL, USA). 22RW1 cells were maintained according to manufacturer's instructions (ATCC) and proteins were extracted as previously described [18].

Samples (20 µg protein) were separated by 7.5 SDS-PAGE under reducing conditions and subsequently transferred to PVDF membranes (Immobilon-P, Millipore, Billerica, MA). The membranes were stained with Ponceau red to confirm equal sample loading and transfer (results not shown). Membranes were then blocked in 5% milk followed by anti-AR antibody incubations; N-20 (diluted 1∶500, Santa Cruz Biotechnology, Santa Cruz, CA) or PG-21 (diluted 1∶1000, Upstate, Lake Placid, NY) in order to detect the ARfl and AR-Vs, and C-19 (Santa Cruz) in order to detect ARfl but not AR-Vs lacking the LBD. Primary antibodies were diluted in 1% milk/PBST and incubated in 4°C over night. The secondary anti-rabbit IgG (Dako, Glostrup, Denmark) antibody (diluted 1∶20 000 in 2.5% milk) was applied after washing in PBST and incubated for 1 h in RT. Protein expression was visualized after extensive washing using the ECL Advanced detection kit (GE Healthcare, Buckinghamshire, UK) and quantified with a ChemiDoc scanner and the Quantity One 4 software (Bio-Rad Laboratories).

### Immunohistochemistry

The bone metastases examined in this study have been previously immunostained for the AR, Ki67 (cell proliferation), activated caspase 3 (apoptotic cells) and PSA antigens (Table 2) following protocols described before [3]. Nuclear AR and cytoplasmic PSA immunostaining were scored according to intensity (0, 1, 2, or 3) and fraction of stained cells (0, 1, 2, 3, or 4). A total score (ranging from 0–12) was obtained by multiplying the staining intensity and fraction scores. A total AR score of 8 or above (median and above) was previously defined as high and found to be associated with a shorter survival after orthopedic metastasis surgery than a AR total score below 8 [3]. A PSA score of 8 and below (median and below) was associated with an unfavorable outcome in CRPC patients [3]. The fractions (%) of Ki67 and active caspase 3 stained tumor cells were previously determined and found not to be associated with outcome in this patient material [3].

**Table 2:** Immunohistochemical data of castration resistant bone metastases stratified according to expression of AR splice variants (AR-V).

	AR-V high^a^^,^ d (n = 10)	Other^a^^,^ d (n = 20)
**AR score** b	12 (8–12)*	8 (0–12)
**PSA score** c	6 (0–12)*	9 (1–12)
**Ki67 index (%)**	19 (11–33)	13 (3.5–44)
**Apoptosis index (%)**	2 (0.3–4.0)	1.3 (0.3–3.3)

Continuous values are given as median (min–max values).

a AR-V high bone metastases were defined to have detectable levels of the AR-V567es transcript and/or the AR-V7 transcript levels in the upper quartile while other CRPC bone metastases had lower AR-V mRNA levels.

b An AR score of 8 or above was defined as high and associated with a particularly short survival after orthopedic surgery [3].

c A PSA score of 8 and below (median and below) was associated with an unfavorable outcome in CRPC patients [3].

*P≤0.05 according to Chi-square test.

d Patients with AR-V high bone metastases were not statistically different from other patients with bone metastases regarding any of the clinical characteristics in Table 1.

### Gene expression analysis

Two-hundred-fifty (250) ng of total RNA per sample was used for cRNA production by the Illumina TotalPrep RNA amplification kit (Ambion Inc, St. Austin, TX, USA) according to the provided protocol. The quality of cRNA was evaluated using the RNA 6000 pico kit (Agilent Technologies) and the Agilent 2100 Bioanalyzer (Agilent Technologies). A total of 750 ng cRNA was used for hybridization to a human HT12-v3 Illumina Beadchip gene expression array (Illumina, San Diego, CA, USA), including 48803 probes and 37846 annotated genes, according to the manufacturer's protocol. The arrays were scanned and fluorescence signals obtained using the Illumina Bead Array Reader (Illumina, San Diego, CA, USA). Array data analysis was performed with GenomeStudio software (version 2009.1, Illumina). Samples were normalized by the cubic spline algorithm and probes with all signals lower than two-times the mean background levels were excluded from the analysis. Differentially expressed genes were identified with the Mann-Whitney differential expression algorithm (*P*<0.05) and defined to have a fold change in-between groups greater than 1.5. Gene ontology analysis was done with the Metacore software (GeneGoInc. St. Joseph, MI, USA).

### Statistical analysis

Correlations between variables were analyzed using the Spearman rank test. Groups were compared using the Mann-Whitney U test for continuous variables and the Chi-square test for categorical variables. Kaplan-Meier survival analysis was performed with death of PC as event and follow-up time as time between metastasis surgery and the latest follow-up examination. Statistical analyses were performed using the Statistical Package for the Social Sciences, SPSS 17.0 software (SPSS, Inc, Chicago, USA). A P-value less or equal to 0.05 was considered statistically significant.

## Results

### High mRNA levels of AR splice variants in CRPC bone metastases

Transcripts for the normal ARfl and the most abundant AR splice variants known so far [16]; AR-V1, AR-V7, and AR-V567es, were detected and their levels quantified in non-malignant and GS7-8 tissue parts of primary prostate tumors and in bone metastases samples, using real-time RT-PCR analysis. As shown in Table 3, the ARfl, AR-V1, and AR-V7 transcripts were detected in most of the non-malignant, primary tumor, and metastases samples examined, while the AR-V567es transcript was detected in 7 (23%) of CRPC bone metastases only.

**Table 3:** Detection of the full length androgen receptor (ARfl) RNA transcript and AR splice variants in prostate tissue and bone metastases.

AR variant	Non-malignant prostate tissue (n = 13)	Primary prostate tumors (n = 13)	Hormone-naïve bone metastases (n = 10)	CRPC bone metastases (n = 30)
**ARfl**	100%	92%	100%	100%
**AR-V7** a	85%	77%	80%	100%
**AR-V1** a	54%	69%	90%	100%
**AR-V567es** b	0%	0%	0%	23%

a AR variants V1 and V7 [14].

b AR variant V567es [16].

**Figure 1:**
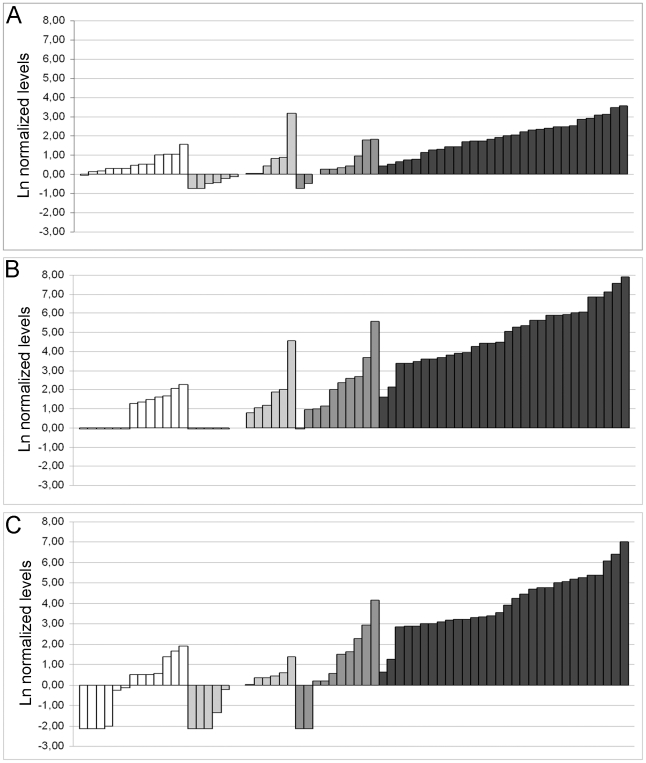
Normalized mRNA levels of the A) full length androgen receptor (ARfl), B) AR-V1 [**14**], and C) AR-V7 [**14**] in a total of 66 tissue samples from prostate cancer patients. All mRNA levels were adjusted for corresponding housekeeping gene (RPL13a) mRNA levels, normalized to the median value for the primary tumors samples, and transformed by the natural logarithm. Levels in non-malignant prostate tissue (*n* = 13), primary prostate tumor (*n* = 13), hormone-naïve bone metastases (*n* = 10), and castration-resistant bone metastases (*n* = 30) samples are shown in white, light grey, dark grey, and black, respectively. Undetectable levels are not indicated, but represented by columns symbolizing the lowest measurable level in RT-PCR analysis of each transcript variant.

There was a tendency for higher levels of ARfl, AR-V1, and AR-V7 mRNA in HN bone metastases than in primary PC tissue, but the differences were non-significant (Figure 1). Clearly elevated ARfl, AR-V1 and AR-V7 transcript levels were however seen in the CRPC bone metastases, which showed 8, 44, respectively 120 times higher median levels than the HN metastases (*P*≤0.001, Figure 1). The ARfl, AR-V1, and AR-V7 mRNA levels in the non-malignant prostate samples were comparable to levels in the primary prostate tumors (Figure 1).

### Expression of LBD-truncated AR protein variants in CRPC bone metastases

**Figure 2:**
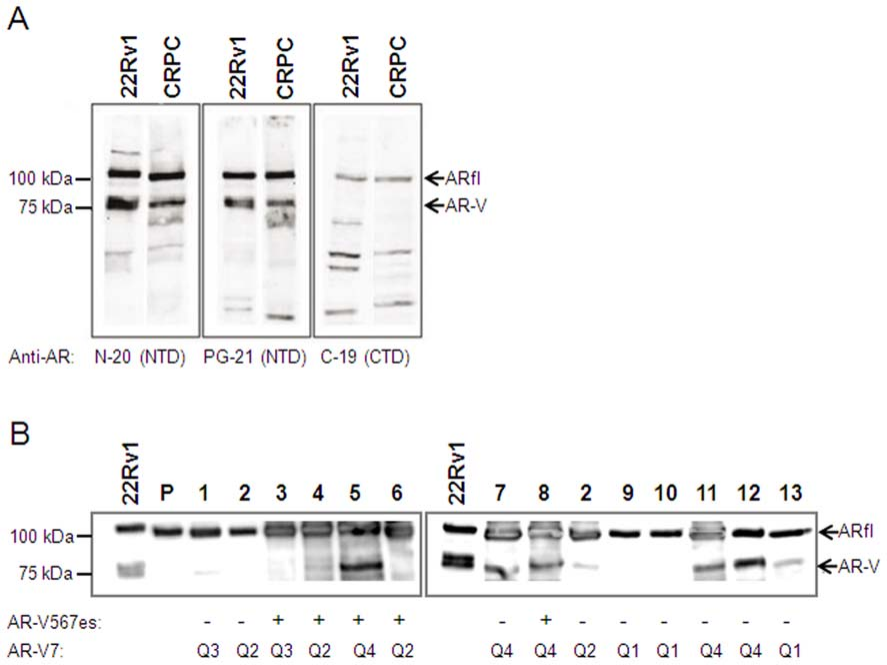
Western blot analysis of the full length androgen receptor (ARfl) and ligand binding domain (LBD) truncated AR variants in castration resistant prostate cancer (CRPC) bone metastasis samples. A) Antibodies (N-20 and PG-21) targeting the N-terminal domain (NTD) of the AR detected the ARfl and AR variants (AR-V; presumingly AR-V1, AR-V7, and/or AR-V567es). Antibody C-19 targeting the LBD detected the ARfl of ∼110 kDa, but not the LBD-truncated AR variants of approximately ∼80 kDa. B) Analysis of CRPC bone metastases representing samples with high levels of AR-V7 mRNA (levels in the highest quartile, Q4, according to RT-PCR analysis), as well as samples with AR-V7 mRNA levels in Q1, Q2, and Q3, by using the N-20 antibody. 22Rv1 cell extract served as positive control for ARfl and ∼80 kDa AR variants and a primary prostate cancer sample (P) served as negative control for the AR variants.

Proteins levels of AR-Vs were examined using Western blot analysis of 13 samples of CRPC bone metastases randomly selected to represent cases with high AR-V7 mRNA levels (levels in the upper quartile, Q4), cases with low and intermediate levels of AR-V7 mRNA (levels in the lowest; Q1, and intermediate; Q2–Q3, quartiles respectively) as well as detectable/non-detectable levels of AR-V567es mRNA (Figure 2). The 22Rv1 cell line known to express the AR-V7 variant [13], but also additional splice variants [15] served as a positive control. A primary prostate sample with undetectable AR-V7 mRNA served as a negative control (Figure 2). Protein bands with the expected molecular weights of about 110 kDa (ARfl) and 80 kDa (presumingly AR-V1, AR-V7 or -V567es) were detected in CRPC bone metastases, by antibodies targeting the N-terminal domain of the AR, while the 80 kDa AR-Vs were not detected using the antibody targeting the C-terminal LBD domain (Figure 2A). The most intense 80 kDa bands were detected in samples with Q4 AR-V7 mRNA levels, while CRPC bone metastases with lower AR-V7 mRNA levels showed lower to undetectable levels regardless of corresponding AR-V567es mRNA levels (Figure 2B). Based on blot intensities, the AR variant proteins constituted in median 32% of the ARfl protein level (range 0 to 95%, *n* = 13, data not shown). This was in contrast to corresponding ratios on the RNA level where the AR-V7 and AR-V567 transcripts represented in median only 0.4% (range 0.03 to 7%, data not shown) and 1.0% (range 0.3 to 1.2%, data not shown), respectively, of the ARfl mRNA level, according to difference in RT-PCR ct levels. Our results thus indicate that AR-Vs lacking the LBD domain are possible post-transcriptionally stabilized in CRPC bone metastases in relation to the ARfl.

### Expression of AR-V7 and AR-V567es mRNA is associated with a poor prognosis

The AR-V1, AR-V7, and ARfl mRNA levels were correlated in all samples examined (data not shown, *n* = 66) and in the sub-group of CRPC bone metastases (AR-V1 vs. AR-V7; *Rs* = 0.91, *P*<0.001, AR-V1 vs. ARfl; *Rs* = 0.47, *P*<0.01, and AR-V7 vs. ARfl; *Rs* = 0.58, *P*<0.001, *n* = 30). Bone metastases with detectable AR-V567es mRNA had about 3-fold higher ARfl median mRNA level than the other CRPC metastases (*P* = 0.004).

**Figure 3:**
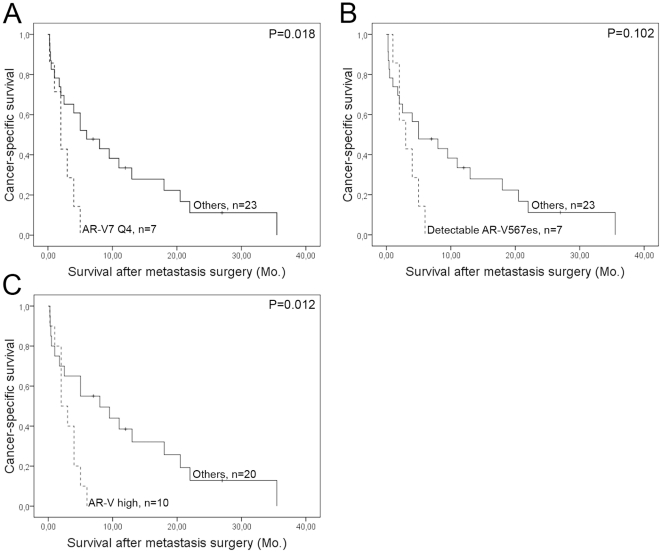
Kaplan-Meier analysis of androgen receptor (AR) variant transcript levels in bone metastasis samples from castration-resistant prostate cancer (CRPC) patients versus cancer-specific survival after metastasis surgery. Patients with A) AR-V7 [14] mRNA levels in the highest quartile (Q4), B) detectable AR-V567es [16] mRNA levels, and C) detectable AR-V567 and/or AR-V7 Q4 (AR-V high) mRNA levels had shorter survival than other patients with CRPC disease.

Patients with AR-V7 mRNA levels in Q4 had significantly shorter cancer-specific survival after metastasis surgery than the other CRPC patients and a similar outcome was seen for patients with detectable levels of the AR-V567es in comparison with the rest (Figure 3A–B). Ten patients with CRPC bone metastases were either included in the AR-V7 Q4 sub-group and/or had detectable levels of the AR-V567es, and are further referred to as AR-V high patients. The AR-V high patients had a median survival time after metastasis surgery of only 2 months as compared with 8 months for the rest of the CRPC patients (Figure 3C). No association was found between ARfl or AR-V1 mRNA levels and survival after metastasis surgery (data not shown).

### Metastases with high levels of the AR splice variants show intense nuclear AR immunostaining

As we have previously found that high AR immunostaining scores were associated with a particularly short survival after metastasis surgery and now that AR-V high patients had a poor prognosis, we wanted to examine if high AR staining score was associated with levels of AR-Vs. The AR-V high metastases all had high AR staining scores in comparison with other CRPC bone metastases which showed variable staining ranging from weak to strong (Table 2, *P* = 0.02).

Furthermore, AR-V high metastases showed significantly lower PSA immunostaining score (Table 2, *P* = 0.038) and tendencies of having higher fractions of proliferating and apoptotic tumor epithelial cells (*P* = 0.12 and *P* = 0.085, respectively) than other CRPC metastases (Table 2).

### Expression of AR splice variants is associated with disturbed cell cycle control

**Figure 4:**
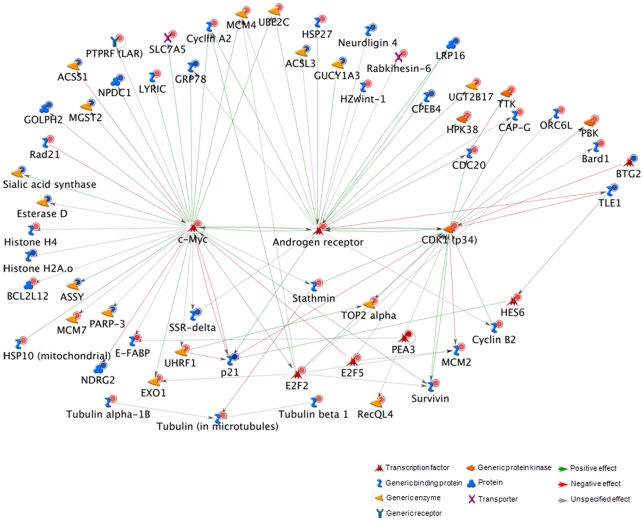
Network of directly interacting gene products of differentially expressed genes between the AR-V high (detectable AR-V567 and/or AR-V7 in the highest quartile, Q4) and other castration-resistance prostate cancer (CRPC) bone metastases (*P*≤0.05 and fold change ≥1.5), generated by Metacore™ (Genego Inc, USA) bioinformatics analysis. Red circles indicate higher and blue circles indicate lower transcript levels in the AR-V high than in other CRPC bone metastases. Note that many of the differentiating gene products are directly interacting via AR, C-MYC and CDK1.

The gene expression profile of the sub-group of AR-V high bone metastases was compared to the profile of other CRPC bone metastases, by using an Illumina based cDNA array including 48803 gene probes. About 17700 probes were defined to give signals above background in the PC bone metastases. One hundred and ninety-five (195) probes were differentially expressed between the AR-V high bone metastases and other CRPC bone metastases according to a fold-change of at least 1.5 in-between groups (*P*<0.05) and further included in ontology analysis for evaluation of enriched processes. Many (60) of the differentiating gene products were proposed to be directly interacting via the AR, C-MYC, and CDK1 proteins (Figure 4 and Table S1). C-MYC and CDK1 are two key regulators of the cell cycle and, accordingly, the top 5 enriched process networks found in AR-V high bone metastases were; Cell cycle Core, Cell cycle S phase, Cell cycle Mitosis, Cytoskeleton spindle microtubules and Cell cycle G2-M. C-MYC and CDK1 are also pro-survival factors inhibiting apoptosis and in addition we found increased transcript levels of the anti-apoptotic proteins survivin and BCL2L12 in the AR-V high compared to the other CRPC bone metastases (Figure 4). Notably, however, levels of other key regulators of apoptosis, such as death receptors, BAX, BCL2, and caspases were not significantly changed and, furthermore, apoptosis-related process networks were not significantly enriched in our data (data not shown). Several gene transcripts known to be positively regulated by the AR were found at high levels in the AR-V high metastases, such as CDK1, CYCLINA2, HSP27, C-MYC, UGT2B17, CDC20, UBE2C, while others such as KLK3 (PSA), KLK2, NKX3-1, FKBP5 and TMPRSS2 were not (Figure 4).

## Discussion

This paper describes, for the first time, the expression pattern of the LBD-truncated AR splice variants AR-V1, AR-V7 [14] and AR-V567es [16] in PC bone metastases in patients and, furthermore, that levels of those variants are increased in CRPC bone metastases and that expression of the AR-V567es and/or very high levels of the AR-V7 is found in individuals with particularly poor prognosis.

Several structurally different, constitutively active AR-Vs have been identified so far in clinical PC samples [10], [11], [13], [14], [16], [17], and we here show enrichment of LBD-truncated AR splice variants in PC bone metastases. In contrast to Sun and co-workers, who found the AR-V567es to be the most abundant splice variant examined [16], we found the AR-V7 to be more commonly expressed than the AR-V567es in CRPC. This disparity could possibly be technically explained by different sensitivities of the RT-PCR techniques used, but could possibly also reflect heterogeneities in AR splice variant expression between different tissue origins as we examined CRPC in bone while Sun et al included CRPC from various sites. We also importantly noted that several of the examined CRPC bone metastases (AR-V high cases) expressed LBD-truncated AR proteins at levels comparable to the ARfl protein levels, even though the corresponding AR variant transcripts were found at relatively much lower levels than the ARfl mRNA. A post-transcriptional stabilization of AR splice variants in relation to the ARfl in selective CRPC bone metastases is therefore plausible, although the mechanism for this is not known.

The poor prognosis of patients with CRPC metastases expressing the AR-V567es and/or high levels of AR-V7 transcripts are probably related to the postulated constitutive activity of these variants. The AR-V567es contains the nucleus localization sequence (NLS) of the hinge region in exon 4 [16] and the AR-V7, in contrast to AR-V1, have been postulated to contain a NLS in its cryptic exon [14]. Accordingly, the AR-V7 and AR-V567es have been shown to translocate into the nucleus, bind to AR responsive elements, and activate/suppress gene transcription *in vitro* without the need for ligand binding [14], [16], [17]. Interestingly, Watson and co-worker recently showed an instant increase of AR-V7 and AR-V1 mRNA levels in xenograft models for PC after castration, as well as a decrease after androgen supplementation, indicating that some AR-Vs may be directly regulated by androgens and thus probably induced in patients shortly after castration therapy [17]. It is tempting to speculate that those and other AR-Vs will be selected for also during the development of castration-resistant disease and thereby contribute to relapse from therapies aiming to reduce steroid levels or ligand binding to the normal AR. Accordingly, we found higher levels of the AR splice variants in CRPC than in HN bone metastases. The AR-V1 and AR-V7 transcripts were however detected also in a substantial part of the non-malignant and malignant radical prostatectomy specimens, although at a lower level than in the bone metastases, and others have shown that levels of AR-V7 (AR3) in primary prostate tumors were predictable for outcome after radical prostatectomy, with a shorter time to chemical relapse in individuals with higher levels [13], [14]. The reason to this is not known, but indicates that constitutively active AR-Vs could be part of the normal prostate physiology and that a selection of those variants occurs during PC progression.

The enrichment of structurally different, constitutively active AR-Vs during the development of CRPC metastases points out complex and heterogeneous molecular events which by difference means seem to ensure AR activation in the absence of testicular androgens. Patients expressing constitutively active AR-Vs will in the long-term probably not benefit from anti-androgen therapies aiming to reduce steroid synthesis, such as surgical castration, LHRH analogs, or the newly introduced agent abiraterone that inhibits CYP17 and thereby steroid synthesis not only in testis but also in the adrenal glands and in tumor tissue [19], [20]. Surprisingly, the novel AR antagonist MDV3100 targeting the LBD of the ARfl and currently in clinical trials for CRPC [21], [22] were found to inhibit castration-resistant growth induced by the expression of constitutive AR-V7 in an animal model for PC [17]. This is encouraging as novel AR antagonists inhibiting receptor translocation into the nucleus thus may have therapeutic effects also in patients expressing constitutively active AR-Vs. Not all CRPC patients do however respond to the abiraterone and MDV3100 drugs, and even those who do subsequently relapse within a few months [20], [22]. If this kind of therapy resistance is related to expression of constitutively active AR-Vs in bone metastases (the primary target for the therapy) is currently not known and cannot be determined until clinical studies starts to include biopsies from bone metastases. Furthermore, therapeutic agents inhibiting those AR-Vs or their down-stream effects need to be developed [23], [24]. Another possible explanation for resistance to castration therapy is the presence of AR negative tumor cells, and thus AR by-pass during tumor progression [25]. Complete lack of AR expression is found only in a small sub-population of CRPC bone metastases, but AR negative tumor cells scattered among the positive are found in most CRPC bone metastases [3], and thus emphasis the need also for therapies not relying only on a functional AR.

In the clinical samples examined in the present study, detectable levels of the AR-V567es and/or high levels of the AR-V7 transcripts were associated with deregulated levels of known AR-controlled genes, but specifically not of some classical androgen regulated genes such as KLK3 (PSA), TMPRSS2, and FKBP5 which were induced by over-expression of AR-V7 or AR-V567es in LNCaP cells *in vitro* [14], [16]. Accordingly, the AR-V high cases showed less PSA immunostaining than other CRPC metastases. Instead we found gene transcript levels which differentiated AR-V high bone metastases from other CRPC bone metastases by indicating high C-MYC and CDK1 activity, and a disturbed cell cycle control regarding G1-S as well as G2-M transition despite a non-significant increase in Ki67 labeling. The development of CRPC has been characterized by an unbalanced proliferation to apoptosis ratio and resistance to apoptotic stimuli [26], [27]. In the late stage of disease examined in this study we did not however, with a few exceptions, find any clear-cut signs of altered expression of apoptosis-regulating genes or a further deregulation of the apoptotic process in AR-V high compared to other CRPC bone metastases. Based on our data, we are not able to discriminate if the disturbed cell cycle control found in the AR-V high bone metastases is truly related to the expression of the AR-Vs, the ARfl, or just associated with the particularly advanced disease in those CRPC cases. Nevertheless, a further understanding of biological processes deregulated in cases with high expression of constitutively AR-Vs could be useful in the development of novel therapies for CRPC. For example, the C-MYC protein could be a therapeutic target in selective patients with PC as already suggested [28].

In conclusion, LBD-truncated AR-Vs are frequently expressed in bone metastases, with higher levels in CRPC than in HN cases. Furthermore, transcript levels of the constitutively active AR-V7 and AR-V567es seem to be associated with high nuclear AR immunostaining scores, disturbed cell cycle control, and particularly poor prognosis within the group of CRPC patients. This conclusion is however based on a limited number of patients and results must therefore be verified in other studies. If the results hold true patients expressing high levels of AR-V7 and AR-V567es probably will need to be treated with drugs acting on or down-stream of the AR-Vs rather than with drugs inhibiting androgen synthesis.

## Supporting Information

Table S1 Differentially expressed genes^a^ between the AR-V high^b^ group and the other CRPC bone metastases.
(DOC)
