# Supplementary material for: Expression of Androgen Receptor Splice Variants in Prostate Cancer Bone Metastases is Associated with Castration-Resistance and Short Survival
Source: PLoS One. 2011 Apr 28;6(4):e19059. doi: 10.1371/journal.pone.0019059 (PMC3084247; doi:10.1371/journal.pone.0019059)
Supplement: Table S1 — Differentially expressed genesa between the AR-V highb group and the other CRPC bone metastases. (DOC) [file pone.0019059.s001.doc]

**Table S1.** Differentially expressed genes^a^ between the AR-V high^b^ group and the other CRPC bone metastases.

| **Gene** | **Network symbol** | **Fold change** | **P-value** | **Description** |
| --- | --- | --- | --- | --- |
| *ETV4* | PEA3 | 6.7 | 0.03 | ets variant 4 |
| *FABP5* | E-FABP | 3.3 | 0.03 | fatty acid binding protein 5 (psoriasis-associated) |
| *HES6* | HES6 | 2.5 | 0.02 | hairy and enhancer of split 6 (Drosophila) |
| *UGT2B17* | UGT2B17 | 2.4 | 0.02 | UDP glucuronosyltransferase 2 family. polypeptide B17 |
| *AR* | Androgen receptor | 2.2 | 0.002 | androgen receptor |
| *BARD1* | BARD1 | 2 | 0.04 | BRCA1 associated RING domain 1 |
| *CDC2* | CDK1 (p34) | 1.8 | 0.01 | cell division cycle 2. G1 to S and G2 to M |
| *TOP2A* | TOP2 alpha | 1.8 | 0.05 | topoisomerase (DNA) II alpha 170kDa |
| *CDC20* | CDC20 | 1.8 | 0.03 | cell division cycle 20 homolog (S. cerevisiae) |
| *UBE2C* | UBE2C | 1.8 | 0.01 | ubiquitin-conjugating enzyme E2C |
| *SLC7A5* | SLC7A5 | 1.7 | 0.04 | solute carrier family 7 (cationic amino acid transporter. y+ system) |
| *E2F2* | E2F2 | 1.7 | 0.02 | E2F transcription factor 2 |
| *MCM7* | MCM7 | 1.7 | 0.0004 | minichromosome maintenance complex component 7 |
| *BIRC5* | Survivin | 1.7 | 0.04 | baculoviral IAP repeat-containing 5 |
| *CCNB2* | Cyclin B2 | 1.7 | 0.02 | cyclin B2 |
| *HSPB1* | HSP27 | 1.7 | 0.02 | heat shock 27kDa protein 1 |
| *TTK* | TTK | 1.7 | 0.004 | TTK protein kinase |
| *E2F5* | E2F5 | 1.6 | 0.03 | E2F transcription factor 5. p130-binding |
| *MCM2* | MCM2 | 1.6 | 0.03 | minichromosome maintenance complex component 2 |
| *MYC* | c-MYC | 1.6 | 0.02 | v-myc myelocytomatosis viral oncogene homolog (avian) |
| *CCNA2* | Cyclin A2 | 1.6 | 0.02 | cyclin A2 |
| *NCAPG* | CAP-G | 1.6 | 0.01 | non-SMC condensin I complex. subunit G |
| *BCL2L12* | BCL2L12 | 1.6 | 0.002 | BCL2-like 12 (proline rich) |
| *MELK* | HPK38 | 1.6 | 0.04 | maternal embryonic leucine zipper kinase |
| *MCM4* | MCM4 | 1.6 | 0.04 | minichromosome maintenance complex component 4 |
| *TUBB* | Tubulin beta 1 | 1.6 | 0.002 | tubulin. beta |
| *ZWINT* | HZwint-1 | 1.6 | 0.02 | ZW10 interactor. transcript variant 3 |
| *RECQL4* | RecQL4 | 1.5 | 0.01 | RecQ protein-like 4 |
| *MTDH* | LYRIC | 1.5 | 0.04 | metadherin |
| *STMN1* | Stathmin | 1.5 | 0.03 | stathmin 1/oncoprotein 18 |
| *EXO1* | EXO1 | 1.5 | 0.02 | exonuclease 1 |
| *TUBA1B* | Tubulin-alpha-1B | 1.5 | 0.003 | tubulin. alpha 1b |
| *UHRF1* | UHRF1 | 1.5 | 0.02 | ubiquitin-like with PHD and ring finger domains 1 |
| *KIF20A* | Rabkinesin-6 | 1.5 | 0.04 | kinesin family member 20A |
| *ORC6L* | ORC6L | 1.5 | 0.001 | origin recognition complex. subunit 6 like (yeast) |
| *PTPRF* | PTPRF (LAR) | 1.5 | 0.03 | protein tyrosine phosphatase. receptor type. F |
| *RAD21* | Rad21 | 1.5 | 0.03 | RAD21 homolog (S. pombe) |
| *HIST1H4C* | Histone H4 | 1.5 | 0.03 | histone cluster 1. H4c |
| *HSPE1* | HSP10 | 1.5 | 0.04 | heat shock 10kDa protein 1 (chaperonin 10) |
| *PBK* | PBK | 1.5 | 0.04 | PDZ binding kinase |
| *PARP3* | PARP-3 | -1.5 | 0.02 | poly (ADP-ribose) polymerase family. member 3 |
| *NPDC1* | NPDC1 | -1.5 | 0.002 | neural proliferation. differentiation and control. 1 |
| *TLE1* | TLE1 | -1.5 | 0.02 | transducin-like enhancer of split 1 (E(sp1) homolog. Drosophila) |
| *ACSS1* | ACSS1 | -1.5 | 0.001 | acyl-CoA synthetase short-chain family member 1 |
| *NANS* | Sialic acid synthase | -1.5 | 0.05 | N-acetylneuraminic acid synthase (sialic acid synthase) |
| *SSR4* | SSR-delta | -1.5 | 0.01 | signal sequence receptor delta (translocon-associated protein delta) |
| *CPEB4* | CPEB4 | -1.5 | 0.01 | cytoplasmic polyadenylation element binding protein 4 |
| *GOLM1* | GOLPH2 | -1.5 | 0.03 | golgi membrane protein 1 |
| *NLGN4X* | Neuroligin 4 | -1.5 | 0.04 | neuroligin 4. X-linked |
| *ACSL3* | ACSL3 | -1.5 | 0.04 | acyl-CoA synthetase long-chain family member 3 |
| *NDRG2* | NDRG2 | -1.6 | 0.05 | NDRG family member 2 |
| *MACROD1* | LRP16 | -1.6 | 0.003 | MACRO domain containing 1 |
| *BTG2* | BTG2 | -1.7 | 0.02 | BTG family. member 2 |
| *GUCY1A3* | GUCY1A3 | -1.7 | 0.02 | guanylate cyclase 1. soluble. alpha 3 |
| *HIST2H2AA3* | Histone H2A.o | -1.7 | 0.03 | histone cluster 2. H2aa3 |
| *MGST2* | MGST2 | -1.7 | 0.002 | microsomal glutathione S-transferase 2 |
| *HSPA5* | GRP78 | -1.8 | 0.0002 | heat shock 70kDa protein 5 (glucose-regulated protein. 78kDa) |
| *ASS1* | ASSY | -1.9 | 0.03 | argininosuccinate synthetase 1 |
| *ESD* | Esterase D | -1.9 | 0.01 | formylglutathione hydrolase |
| *CDKN1A* | p21 | -2.3 | 0.01 | cyclin-dependent kinase inhibitor 1A |

^a^ Gene products directly interacting via AR. C-MYC and CDK1 according to network analysis using Metacore^TM^ (Genego Inc. USA) were included. Fold change ≥ 1.5 and p ≤ 0.05. ^b^AR-V high bone metastases were defined to have detectable levels of the AR-V567es transcript and/or the AR-V7 transcript levels in the upper quartile.
